# Parametric Evaluation of Errors Using Isolated Dots for Movement Measurement by Image Cross-Correlation

**DOI:** 10.3390/s18020525

**Published:** 2018-02-09

**Authors:** Belen Ferrer, David Mas

**Affiliations:** 1 Civil Engineering department, University of Alicante, Carretera San Vicente del Raspeig, s/n, 03690 Sant Vicent del Raspeig, Spain; 2 Institute of Physics Applied to Sciences and Technologies, University of Alicante, Carretera San Vicente del Raspeig, s/n, 03690 San Vicente del Raspeig, Spain; david.mas@ua.es

**Keywords:** normalized cross-correlation, subpixel tracking accuracy, simulated speckle, error, movement measurement

## Abstract

Digital Image Correlation (DIC) is a common tool for assessing the movement of objects in a scene. Among others, one of the most popular techniques consists of tracking a dotted texture imitating speckle patterns. In this work, we analyzed the individual dots that form this pattern in order to propose an optimum size, shape, and dynamic range that allows minimizing the tracking error. Tracking was accomplished by using normalized cross-correlation with peak interpolation in order to obtain subpixel accuracy. For the models here used, we show that dot radii of 30–40 px with 150 gray levels are enough to obtain an accurate subpixel tracking resolution. Also, we show that 0.002 px is the performance limit of this technique, being this limit in accordance with the experimentally achievable subpixel limit.

## 1. Introduction

**Figure 1:**
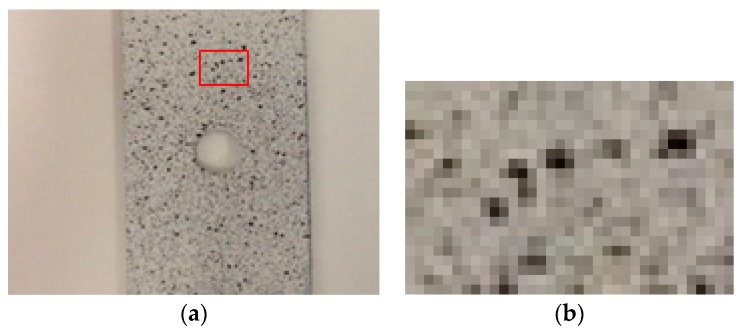
(**a**) Texture resulting after painting with an atomizer on a metallic surface, after [9]; (**b**) detail of the texture with isolated dots.

In the latest years, image correlation has been widely used in a broad spectrum of fields, such as geothecnics [1], structural health monitoring [2], particle velocimetry [3], material characterization [4], or medicine [5], among others. In most of these applications there is not a clear target to follow, so the surface texture is used instead. In some cases, the surface is soft and the texture does not have enough contrast to allow an effective application of tracking methods; therefore, an artificial pattern is painted on the surface. This pattern has to be complex enough to allow the detection of local movements and deformations through the whole specimen under study. Thus, dotted, speckle-like patterns are often used (see Figure 1). These patterns (often called “simulated speckle”) can be easily painted on a surface by using some paint with an atomizer. This easy preparation, together with the uniformity of the points and the high contrast between points and background, makes this pattern suitable for dynamic correlations. In fact, some commercial software has been developed using Digital Image Correlation (DIC) and speckle patterns added or painted in the surface, such as GOMCorrelate [6], Ncorr [7] or GeoPIV [8].

When recording a series of images during the movement of an object with a texture, the details in the surface move together with the object and thus they appear in consecutive different images with the only difference of their position with respect to the image edges. DIC calculation between two consecutive frames provide the relative displacement of one pattern with respect to the previous one, and the movement description can thus be obtained.

Since DIC is defined on discrete sets, the tracking resolution is limited to one pixel, which is not enough for the majority of applications. Subpixel resolution can be achieved by interpolation which is applied either on the images that are being compared prior to calculate the correlation function [10] or on the correlation function itself, in order to increase the accuracy of the maximum location function [11]. The first approach is commonly used for assessing deformations in solid materials since it allows an efficient implementation of deformation mappings. The second one is faster and of easy implementation in non-deforming subsets. It is usually preferred for tracking isolated or sparse objects through image cross-correlation and it is often used in particle tracking and velocimetry.

**Figure 2:**
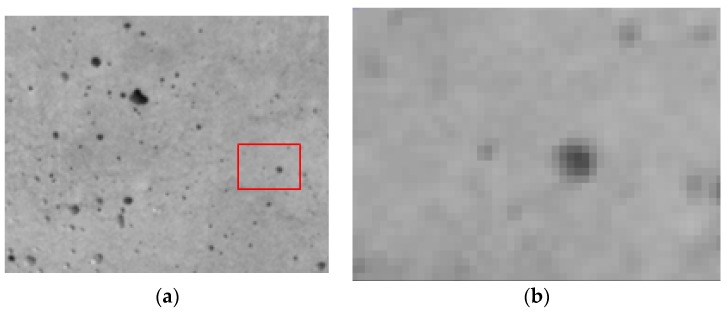
(**a**) Concrete surface pattern; (**b**) enlarged detail of a surface defect. Notice the degraded intensity surrounding the central dot and the low definition of the image at this scale.

In [12], the authors proposed a technique for measuring the deformation in a concrete probe under loading–unloading cycles by tracking the displacements of the surface defects. These defects were due to small bubbles that remained added to the formwork surface during concrete hardening (see Figure 2). The used method consisted in dividing the image in small subsets forming a mosaic so that each of the subsets contained a small number of hollows. The test machine and the temporal resolution of the camera were adjusted in order to allow very subtle changes between consecutive frames. By comparing each subset with the corresponding one in the following frame, the authors determined the relative shift of the bubbles and thus obtained the probe deformation as a function of time. Tracking the concrete surface was done by calculating the normalized cross-correlation between two consecutive subsets and then performing a local peak interpolation in order to achieve subpixel accuracy. The proposal resulted in an accurate and fast method for the analysis of concrete deformation.

In Figure 1b and Figure 2a, one can notice that the textures of the painted speckle and concrete are similar. At a local scale, both textures show a distribution of isolated dots and therefore both textures can be analyzed following similar methods. We would like to underline that this pattern is not only found in simulated speckle or concrete surfaces but also in other materials like granite, aerogels, particles in liquids, or aggregates like terrains.

In [12], we noticed that some characteristics of the single dots, like the object size, the definition, and the brightness, play an important role in the final accuracy of the tracking method. To our knowledge, the specific characteristics of dotted texture images that provide the best results have not yet been analyzed in detail. Recently, a related work from LePage has analyzed the best way of performing the painting of speckle. There, it is shown that using a white paint as a background with black spots on it gives a better accuracy than the opposite, because of the best hiding power of the black paint versus the white paint [13]. From our point of view, this result could also be linked to the number of gray levels included in each case.

However, the dots size and the intensity gradient surrounding each point, which is connected to the object definition (see Figure 2b), were not conveniently analyzed.

The point size is linked not only to the way in which a dotted pattern is created, but also to the appearance of the recorded image. Different magnifications may provide different point sizes. Additionally, the image resolution will influence the gradient of intensities that appear around the central point. This gradient is indirectly connected to the dynamic range of the registered image and thus is connected to the expected accuracy of the method [14].

Regarding the errors obtained using artificial speckle images and image correlation, a study by Bornet et al. [15] assessed the errors in the measurement of deformations by using three different speckle sizes among other parameters, such as the subset size and the shape function. However, their results did not show conclusive assessments about the speckle sizes or the intensity gradients that appeared around each pattern point. Other studies focused on the errors due to intensity interpolation [16,17] on undermatched subset shape functions [18,19] or on overmatched shape functions [20,21]. The light intensity influence on errors was analyzed by [22] among other parameters, showing that the error potentially decreased as the mean image intensity increased. The influence of the subset size was studied by [23], concluding that larger subsets improve the tracking error. The same conclusion was given in [24].

Notice that all works cited in the above written paragraph refer to speckle and propose a mathematical analysis based on this particular texture, which has a defined range of sizes and spatial statistics. From our point of view, the parameters defining this texture may change with the optical system. High-resolution sensors with high zooming produce patterns in which the characteristics of the individual spots defining the texture become relevant for the analysis, and thus, other points of view may be necessary for an optimum tracking of the surface.

In this work, we propose the study of the influence of three main parameters defining the individual objects in a dotted pattern: the point size, the gradient of intensities around the point, and the peak luminance. These parameters have been selected since they can be directly connected with the experimental implementation of a tracking experiment. The brightness is directly related to the illumination of the sample, and the intensity gradient around the central dot is linked to the optical system and the charge-coupled device (CCD) resolution. The dot size is determined by the texture, but it can be modified in the image by changing the magnification through the optical system or by changing the distance from the object to the camera. Other parameters that also affect the errors, such as the type of movement registered, the intensity interpolation, the shape functions, the subset size, or the interpolation function for peak refinement, are maintained fixed. Numerical experiments are used in order to permit changing all parameters and performing arbitrarily small subpixel shifts. The final aim of the paper is to find those configurations that would allow the maximum subpixel resolution under ideal circumstances and thus help to proper configure an experimental test.

## 2. Materials and Methods

### 2.1. Object Design

To study the influence of all the above mentioned parameters in the detection of movement using image cross-correlation, different numeric test objects were created. These objects were created so that one can control some of the characteristics that would influence the results, that is, the size of the point, the dynamic range, and the gradient of intensities [25].

Points were here approximated by circles, since the basic point shape is not very far from a circle (see Figure 1 and Figure 2). The design of the object was done by stacking 255 concentric circles of value 1 and diminishing their diameters. In this way, we built a peak with a top plateau value of 255, corresponding with a white color in 8-bits images, which is the most common codification in commercial grayscale cameras. The size of the plateau, defined by the smallest circle (the one on the top of the figure having the highest value) was used as the point size.

**Figure 3:**
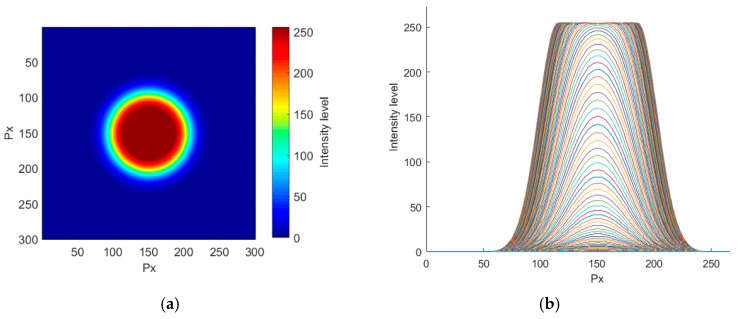
Point design: (**a**) upper view; (**b**) lateral view of its level contour lines.

The radii variation from the first circle in the bottom to the top one represents the gradient of intensities that can be found around the point in the image. This variation was designed as a Gaussian curve (Figure 3). The design of the point in this way allowed the parameters of point size and gradient to be changed independently. The gradient is linked to the number of intensity levels; notice that too stepped gradients will not include all the 256 intensity levels. The width (and slope) of this halo around the central plateau is connected with the definition of sharpness of an object. Narrow halos represent well focused objects with sharp borders, while extended areas may represent a full variety of objects, like objects with a soft profile, affected by diffraction, unfocused, or even undersampled (see Figure 1b and Figure 2b).

Another interesting parameter to be considered in the analysis is the ratio between the area of the upper part and the area occupied by the slope down to the base of the object. To determine the radius of the base, the whole figure was binarized using Otsu’s method [26]. If R is the radius of the point and Rb is the radius of the base, the area ratio is:

(1) $$A_{R}=\frac{\pi R_{b}^{2}-\pi R^{2}}{\pi R^{2}}=\frac{R_{b}^{2}}{R^{2}}-1$$

The reader should notice that the definition of the target was done in positive luminances. The analysis that is presented below can be easily extended to negative images just by taking the complement image, i.e., 255-L, being L the luminance of the original positive dot.

### 2.2. Image Processing

A series of dot images were used as test benches. Each test was composed of a sequence of frames showing a moving dot defined as explained above. Each frame showed the same dot, shifted 0.005 px from the initial position. The full sequence was composed of 200 frames, thus the total displacement was 1 px. The analytical definition of the object given in the previous section allowed to control small and constant displacements between consecutive images. Also, the frame size was fixed to 1500 × 1500 px, thus ensuring that the object representation was accurate [25].

The displacement of the dot was checked through normalized cross-correlation between the original frame and the subsequent frames which contained the shifted object. To this end, we used the *normxcorr2* algorithm implemented in Matlab. Notice that this algorithm requires that the template frame is smaller than the original image. Therefore, the frames with the shifted objects were cropped by removing an external band of 8 pixels in all image sides. No intensity interpolation was done on the images and no shape function was used, i.e., no deformation was allowed between the two images. In order to achieve a subpixel tracking resolution, the correlation peak was interpolated in a 3 × 3 pixels neighborhood area around the maximum, following the ideas described in [11]. The peak interpolation was done by using the first terms of a Taylor polynomic expansion around the peak, resulting in a quadratic surface, and finding the location of the maximum point of this analytic surface. 

Once the complete movement history was obtained, it was compared with the theoretical displacement. The error was defined as the difference between the calculated and the theoretical positions for each one of the 200 steps. Here, we studied the maximum error and the standard deviation of the errors in a full sequence.

## 3. Results and Discussion

**Figure 4:**
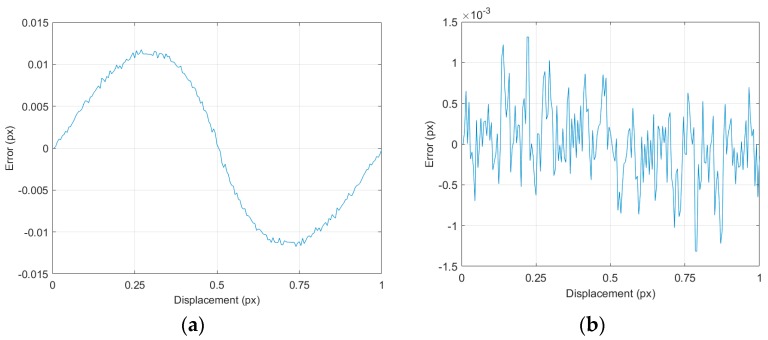
Example of the curve shape in the errors obtained: (**a**) big error, for a point size of 25 px, 43 intensity levels, and area ratio of 0.28, the maximum error is 0.01174 px; (**b**) small error, for a point size of 25 px, 232 intensity levels ,and area ratio of 3.84, the maximum error is 0.001315 px.

For all studied cases, the calculated error showed a rotational symmetry with respect to a displacement of 0.5:

(2) Error(d)=−Error(1−d)

being d the displacement which is defined in the interval [0, 1] pixels. When the error is large, the curve tends to a sinusoidal shape with small oscillations due to error fluctuations, as it is shown in Figure 4a. In this last case, the maximum absolute error could be found at displacements about 0.25 px and 0.75 px. However, as the simulation error decreased, the noise in the curve increased until it completely distorted the smooth shape of the curve, as shown in Figure 4a, and it became similar to the graph shown in Figure 4b. The location of the maximum could now be anywhere, but symmetry expressed in Equation (2) remains true, regardless of the error magnitude. That symmetry is due to the fact that, when a full pixel displacement is accomplished, the displaced object is exactly equivalent to the original one but in a different position.

The behavior shown in Figure 4a is known as peak-locking or bias locking and it is described in [27]. Briefly, this happens because the peak neighborhood in the correlation function is not symmetric. The use of symmetric interpolant functions makes the result (i.e., the maximum of the interpolant function) tend towards the highest values of the original samples neighborhood. Using a non-symmetric interpolant function allows to have non-symmetric surroundings without affecting the location of its maximum.

Although different algorithms have been proposed to compensate this effect, this effect is inherent to the method and thus it always appears [28]. In our case, we decided to use the simplest and most used method (quadratic interpolation), despite the known limitations. Since all the tests were calculated through the same algorithm, the bias affected all of them in the same way. The use of an error reduction algorithm shifted the curves and produced lower errors, but the comparison among different cases remained the same.

### 3.1. Influence of the Point Size

**Figure 5:**
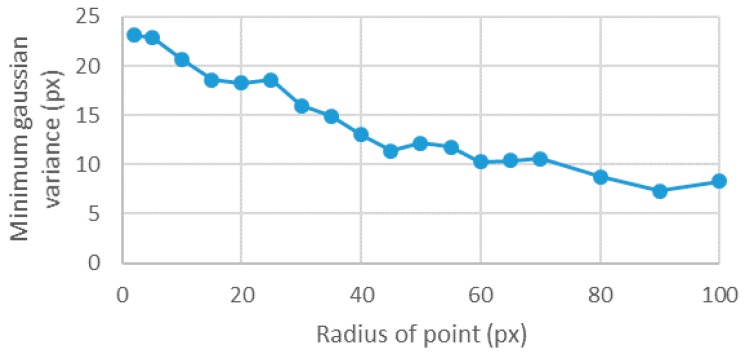
Relation between the point size and the minimum Gaussian variance needed to have 256 intensity levels.

The point size cannot be analyzed as an independent parameter, as it is linked to the gradient and to the number of levels. With large enough point sizes and variances, all 256 intensity levels were present. However, while the point size decreased, for the same variance, the flank width was not enough to include all levels. Therefore, each point size had its own minimum Gaussian variance that allowed the inclusion of all the 256 intensity levels. In general, the minimum variance decreased with the point size, but there were local maxima (Figure 5).

Furthermore, for different sizes, two points of view may be considered with respect to the gradient of intensities surrounding the central point. The first one is to consider the area ratio as fixed, letting the involved number of levels change. The second approach is to fix the dynamic range and let the area ratio change. Both points of view were analyzed in the following sections.

#### 3.1.1. Influence of the Point Size for the Same Area Ratio and Different Dynamic Ranges

**Figure 6:**
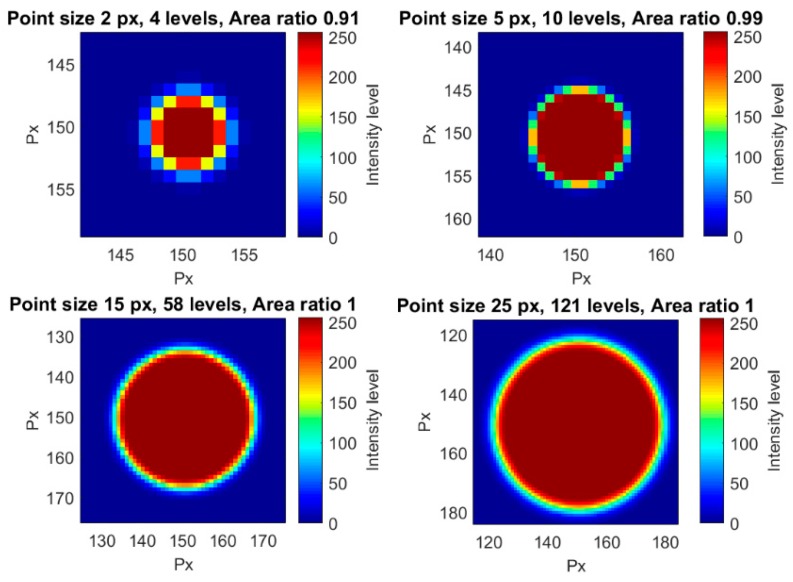
Four different examples of points with different point sizes and an area ratio of 1.

The influence of the point size for a constant area ratio was analyzed in this section. The only way to maintain constant the area ratio for a changing point size is decreasing the number of levels with the point size. That led to poorer halos and point resolutions. In Figure 6, four different point sizes for the same area ratio are shown, as an example. Different visualization sizes were also used in order to clearly show the different point resolutions. For a point size radius of 100 px, a total of 256 levels were obtained, so this was the largest point size in our tests.

**Figure 7:**
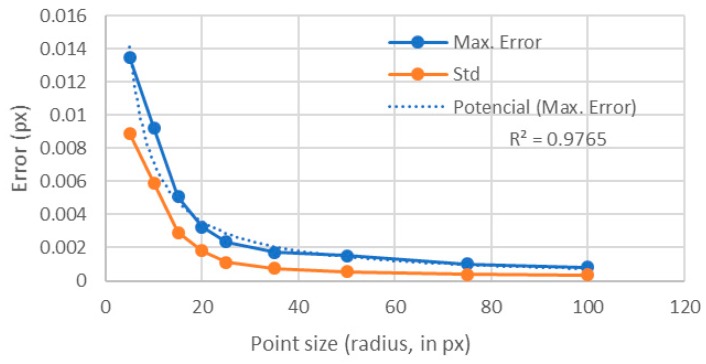
Influence of the point size when the area ratio was set to 1.

**Figure 8:**
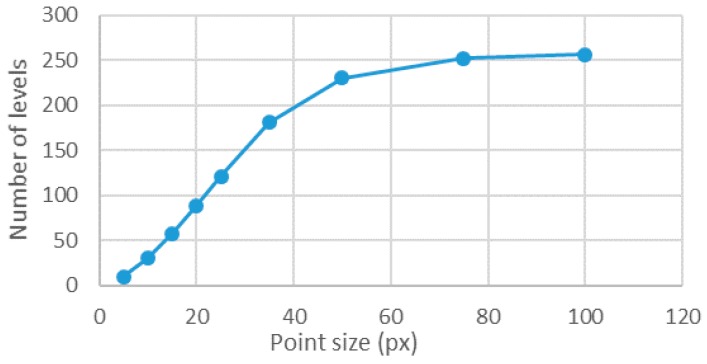
Number of levels involved over point size for an area ratio of 1.

The results showed that the error quickly decreased as the point size increased (Figure 7). This behavior was found both for the maximum error and for the standard deviation. In Figure 8, it is shown that, as the point sizes increased, having a fixed area ratio of 1, the number of gray levels also increased. Therefore, one may question was whether the error was decreasing because of the point size or because of the rise of the levels number [14]. Therefore, in the following section, the number of levels was fixed to 256 and the influence of the point size was analyzed. Of course, the only way to have always 256 levels for different point sizes was to have different area ratios.

#### 3.1.2. Influence of the Point Size for the Same Dynamic Range and Different Area Ratios

**Figure 9:**
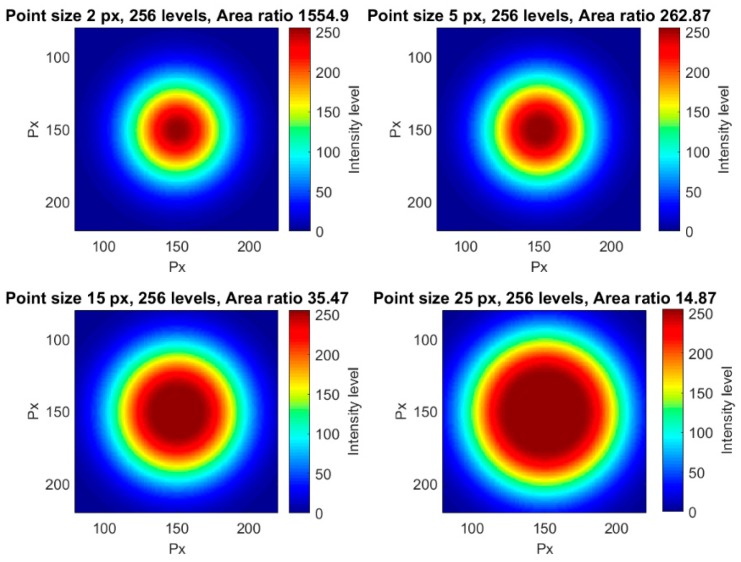
Four different examples of points with different point sizes and the same number of levels.

**Figure 10:**
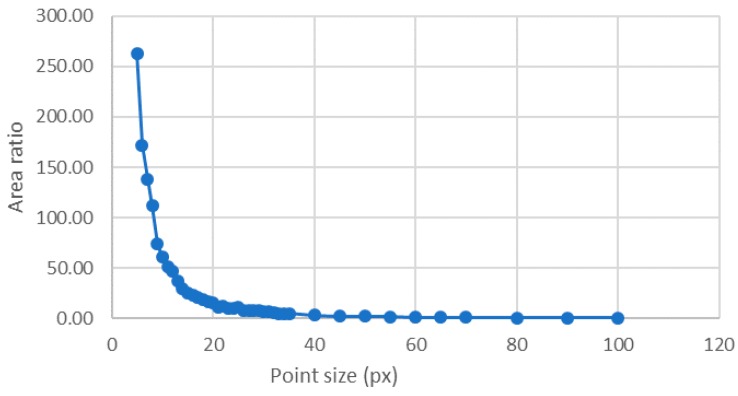
Area ratio value over point size when 256 levels were involved having the minimum possible Gaussian variance, according to Figure 3.

To analyze the point size influence with all possible luminance levels, i.e., 256, different objects were created in which the number of levels was set to 256. In all cases, the variation of the halo width was adjusted to be minimum for the 256 intensity levels. Figure 9 shows some points having different sizes for the same number of gray levels, as examples of all the considered points in this section. In this case, the area ratio decreased while the point size increased (Figure 10).

**Figure 11:**
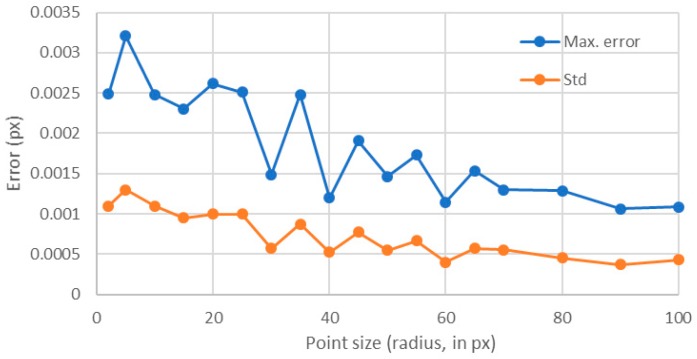
Influence of the point size for 256 intensity levels.

**Figure 12:**
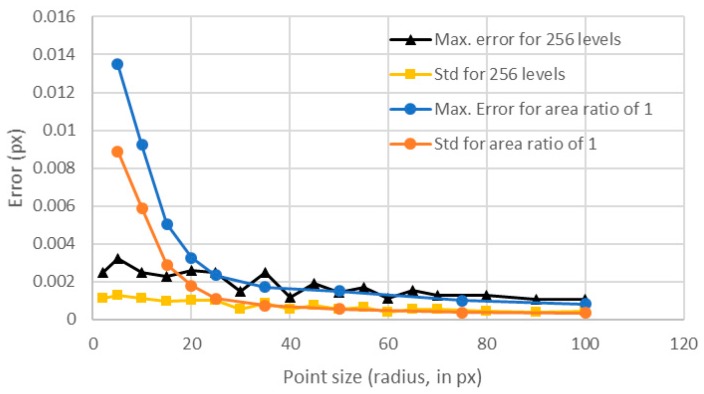
Comparison between the errors obtained for an area ratio of 1 and for 256 levels (superposition of Figure 7 and Figure 11).

Contrary to the previous analyzed case, the calculated error seemed to be slightly decreasing, but with a very noisy behavior (Figure 11). For the standard deviation, this trend was also observed. In Figure 12, we compared these values with those obtained for an area ratio of 1 (Figure 7). One can see that there was a big difference between the errors obtained with a constant area ratio of 1 and those obtained using 256 levels up to a point size of radius of 35 px. This happened because of the reduced number of levels for the points with area ratios of 1. Notice that for points of larger sizes than 35 px, both errors had similar values because for those sizes almost 256 levels were involved, even for the area ratio of 1 (Figure 8).

Therefore, regarding the point size influence on errors, one can conclude that it is mainly due to the number of intensity levels that are included for each point size, and not to the point size itself. Also, notice that the maximum error for an area ratio of 1 could be adjusted to a potential law and that, from a point radius of 35 px, the error did not improve significantly with larger point sizes. Therefore, for an area ratio of 1, the optimal point radius was between 30 and 40 px. Larger sizes may require more complicated and expensive imaging setups and will only result in slight improvements in the errors, thus resulting in high cost-effective methods. Regarding the dynamic range, if 256 levels were involved, the error had a slight but constant decreasing trend with increasing point size values.

### 3.2. Influence of the Gradient

**Figure 13:**
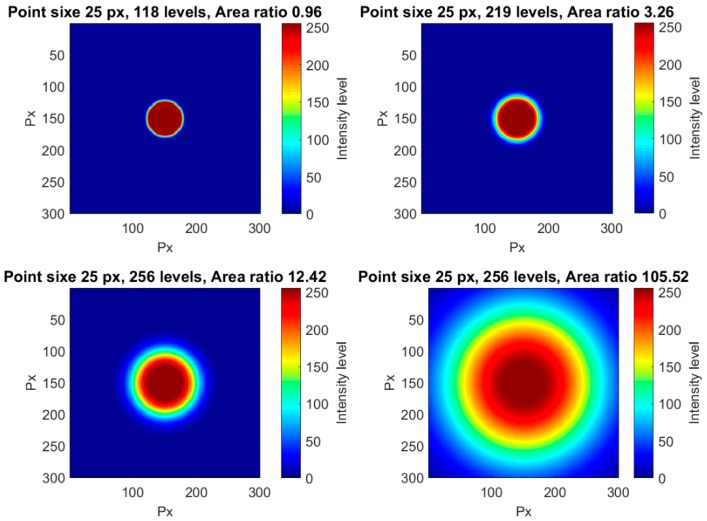
Four different examples of points with the same point size and different surrounding gradients.

The variation in the gradient was analyzed for a fixed point size. A radius of 25 px was selected in order to have a middle error value related to the point size, as explained in the previous section. Increasing the halo radius by means of increasing the variance of the Gaussian increased the number of intensity levels and also the ratio between areas. Therefore, the influence of the gradient error was studied through the area ratio. Figure 13 shows some examples of objects having the same inner area but different surrounding areas.

**Figure 14:**
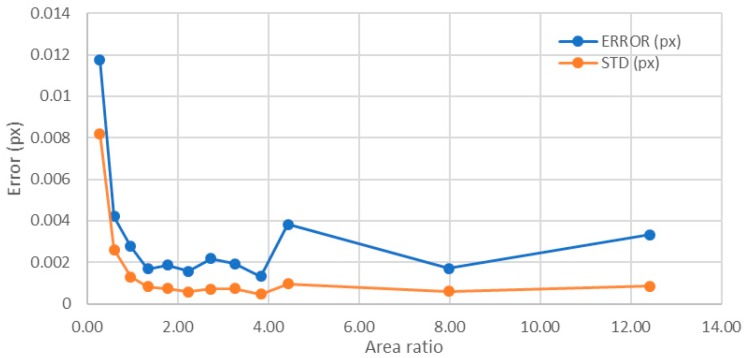
Influence of the area ratio for a point radius of 25 px (from 0 to 256 levels).

For a number of levels below 256, the error decreased quickly with the increasing area ratio, with a singular point in the area ratio of 4.43 that had an error higher than expected (Figure 14). From an area ratio of 1.35 on, the average maximum error was around 0.002 px, and the standard deviation was 0.0007 px. This error stabilization around 0.002 px is in accordance with the above described case and also with the hypothesis in [14].

**Figure 15:**
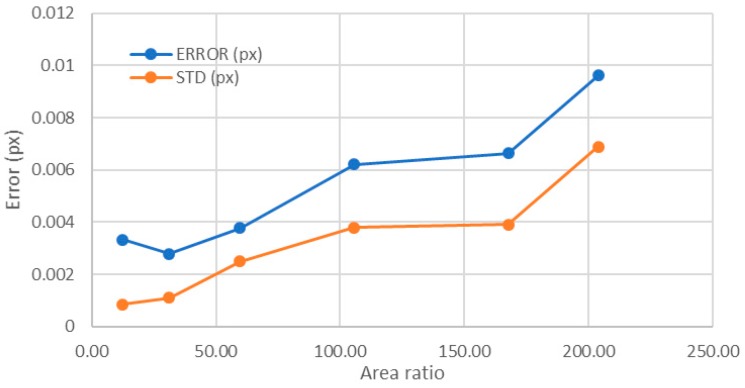
Influence of the area ratio for a point radius of 25 px (all having 256 levels).

For area ratios larger than 12.42, the number of levels was constant and equal to 256. Therefore, the only meaningful representation was for objects above this area ratio in order to avoid the influence of the number of levels in the analysis. In this representation, the behavior changed in comparison with the previous explained cases, and the error increased (Figure 15). Notice that, if the base of the object enlarged while keeping constant the size of the upper part of the point as well as the number of levels, the error increased until reaching a value similar to that obtained having only 43 levels. This means that the parameter that improved the error when the point size was constant was the number of levels in the image and not the area ratio. Therefore, the optimum value of the area ratio (equivalent to the gradient) was between 1.35 and 4. Larger area ratios, in spite of having higher number of levels, did not improve the error obtained.

### 3.3. Influence of the Illumination (Maximum Intensity and Dynamic Range)

**Figure 16:**
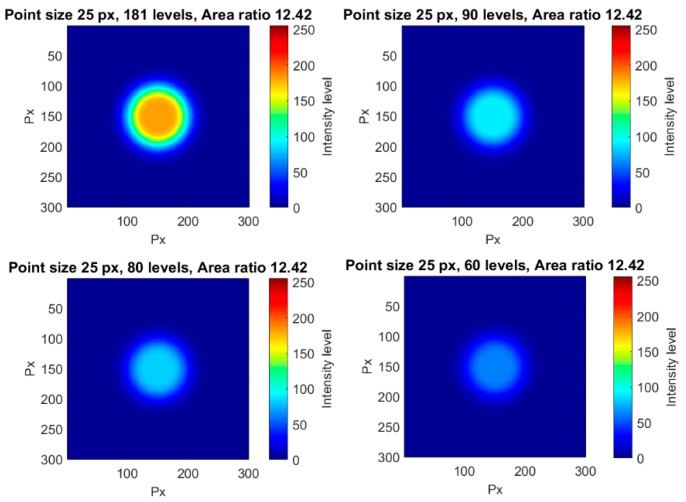
Four different examples of points with the same point size, same surrounding gradient, and different maximum intensity levels.

Different illuminations were simulated here for the same point size and same area ratio. The effect of having more or less illumination was that the maximum value in each point was higher or smaller, respectively. For an optimum illumination, the maximum intensity value would be 256, but, with lower intensities, the maximum value and the number of levels involved may decrease. This effect was simulated by downsizing the whole image intensity with a new maximum, lower than 256. Figure 16 shows some examples of variation in the maximum intensity for the same point size and area ratio.

**Figure 17:**
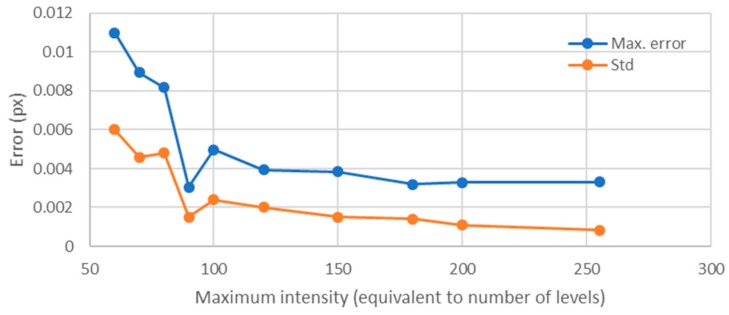
Influence of illumination for a point radius of 25 px and an area ratio of 12.42.

Since the illumination level is linked to the number of intensity levels, the error showed a fast descending trend (Figure 17). As it was observed for the influence of the point size for a constant area ratio, once 150 levels were involved, the error descended very slowly with the increment of the number of levels. This result matched with that obtained by Haddadi et al. [22] in which the error due to luminosity decreased with the rise of the mean intensity values. In our work, as the background had a constant intensity, the increase of the maximum intensity led to an increase of the mean intensity as well.

## 4. Conclusions

In this paper, the errors obtained in measuring a rigid displacement (without deformation or rotation) through image cross-correlation were analyzed. The focus was set in the analysis of one single object that could belong to a more complex dotted texture pattern. The object was designed to have control over its size, both in its higher and lower intensity areas, as well as over the number of levels involved and the maximum current intensity. The patterns used here were similar to those commonly used in DIC, but in our case, the focus was set on the particular elements that formed the structure and not on the texture itself, with their particular links and statistical properties.

The results showed that, when analyzing the individual dots forming the texture, the main parameter that influences the error was the number of levels involved. However, it was also demonstrated that, above 150 levels, increasing the number of the levels did not produce a significant improvement of the error. Regarding the point size, for area ratios close to 1, the optimum value corresponded to a radius between 30 and 40 px. Below these values, the error increased quickly, while, above these values, the error did not improve significantly (Figure 12). For higher area ratios (including all 256 levels), this optimum value was not so clear, because for smaller point sizes the error increase was not so big as in the previous case. The study including all 256 levels was included to analyze separately the influence of each parameter, but, in real images from digital cameras, images showing all the 256 levels around each point were difficult to obtain. Images like those shown in Figure 6 are much common in standard applications than those shown in Figure 9; therefore, we can state, without lack of generality, that the optimum value for the point radius was between 30 and 40 px.

The study of the intensity gradient around the point (measured as the area ratio between the top planar area and the descending sloped ring around it) led to an optimal value of the area ratio of 1.35. Higher intensity gradients between the point and the background than that obtained for an area ratio of 1.35 not only did not improve the results, but worsened them very quickly, while smoother gradients did not affect the error significantly. Regarding the influence on errors of the maximum intensity (linked to the illumination level), the higher was the maximum level, the higher the number of levels involved, and, as a consequence, the error decreased. However, as for the rest of parameters, once reached 150 levels, the increase of the maximum value (or, equivalently, of the number of levels) did not improve the results.

The reference values here obtained for the different parameters may be used to design the measurement procedure and the illumination setup in order to have the maximum accuracy in the results at a minimum cost.
